# Subject-specific modeling of response to physical stress via hypothalamic-pituitary-adrenal and sympathoadrenal axes

**DOI:** 10.1371/journal.pone.0344981

**Published:** 2026-03-24

**Authors:** Helen A. Harris, David M. Chan, Laura Ellwein Fix, Benjamin D. Nicholson, Edmund O. Acevedo

**Affiliations:** 1 Department of Mathematics & Applied Mathematics, Virginia Commonwealth University, Richmond, Virginia, United States of America; 2 Department of Emergency Medicine, Virginia Commonwealth University, Richmond, Virginia, United States of America; 3 Department of Kinesiology and Health Sciences, Virginia Commonwealth University, Richmond, Virginia, United States of America; Northwest Institute of Plateau Biology Chinese Academy of Sciences, CHINA

## Abstract

The two main pathways for hormonal stress response are the hypothalamic-pituitary- adrenal (HPA) axis and the sympathoadrenal (SA) axis. The HPA axis produces and secretes cortisol, while the SA axis produces and secretes the fast-acting catecholamines, epinephrine and norepinephrine, which in turn stimulate cortisol. Since it is difficult to consistently measure or monitor their concentrations in plasma, mathematical modeling of the catecholamines and their connection to cortisol can provide more information about the acute stress response. Previous mathematical models have simulated the dynamics of the HPA axis, but a model of the SA axis has not been created nor one with the combined effects of the HPA and SA axes. We propose an extension of Bangsgaard and Ottesen’s differential equation-based HPA axis model that includes the SA axis [1]. We performed sensitivity analysis using Morris screening and estimated model parameters using constrained optimization with respect to time series data of cortisol and catecholamine dynamics under acute physical stress. After subject-specific parameter estimation, the proposed model that includes both the HPA and SA axes shows qualitative agreement with the collected data.

## 1. Introduction

Cortisol is the main hormone associated with physiological stress response in humans. Dysregulation of cortisol is associated with many adverse chronic health conditions including Cushing’s disease, adrenal insufficiency, diabetes, depression, and Alzheimer’s disease [1–5]. Implementation of effective ways to measure and predict both short- and long-term cortisol levels remains a critical component of diagnosing and treating such conditions.

**Fig 1:**
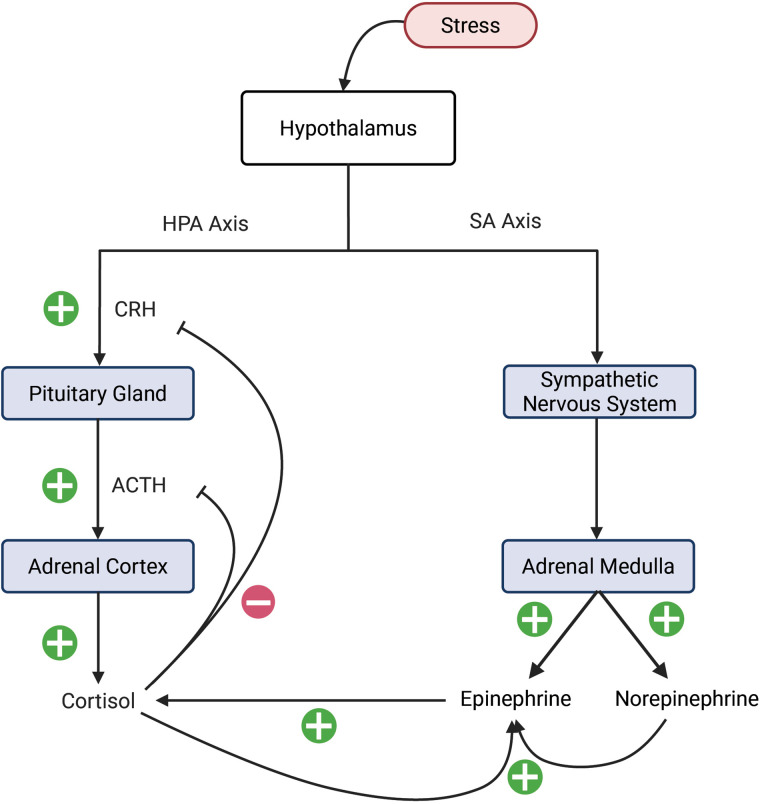
Dynamics of the HPA and SA axes. Process of cortisol production and secretion via the HPA axis (left) and catecholamine production and secretion via the SA axis (right).

There are two main pathways that mediate the body’s hormonal stress response (see Fig 1). First, the hypothalamic-pituitary-adrenal (HPA) axis, which is made up of the hypothalamus, pituitary gland, and adrenal cortex, produces cortisol. The HPA axis functions on a 24-hour circadian cycle to increase cortisol at times when bodily arousal is necessary for daily functions. In healthy subjects, cortisol secretion will be maximal around the time of waking and near zero around the onset of sleep [6]. In addition to circadian oscillations, cortisol also exhibits shorter ultradian oscillations throughout each day [1,7].

The process of cortisol production starts when the hypothalamus responds to environmental stimulus by secreting corticotropin-releasing hormone (CRH) into the bloodstream. CRH triggers the synthesis and release of adrenocorticotropic hormone (ACTH) from the pituitary gland, which then travels to the adrenal cortex to signal the production of cortisol for release into the bloodstream [1,7,8]. Once it is released, Cortisol exerts negative feedback on CRH and ACTH [8].

While the HPA axis regulates the body’s long-term stress response, a second pathway known as the sympathoadrenal (SA) axis regulates the short-term stress response, often referred to as the “fight-or-flight” response [9]. The SA axis is formed by the hypothalamus and adrenal medulla, which communicate via the sympathetic nerves, producing the catecholamines: epinephrine and norepinephrine [8–10]. It has been observed that norepinephrine is converted into epinephrine via the Phenylethanolamine N-methyltransferase (PNMT) enzyme [11]. The prevalence of this enzyme in the body increases with higher concentrations of cortisol [11].

A greater understanding of the dynamics of both sets of hormones can be achieved with a computational model that simulates experimental data. Previous modeling efforts have shown dynamics of cortisol under various conditions, but so far have only focused on the HPA axis. Both Terry et al. and Gupta et al. included a state variable representing the concentration of glucocorticoid receptors in addition to the three main products of the HPA axis [7,12]. Bangsgaard and Ottesen took a patient-specific approach to model parameterization with a focus on differentiating between HPA axis activity for depressed and non-depressed subjects [1]. No previous models have sought to mathematically describe the SA axis nor the concurrent responses of both the HPA and SA axes to stress.

To address the gaps in knowledge about their short- and long-term dynamics during acute stress events, we propose a model that includes the effects of both the HPA and SA axes. This model incorporates state variables representing plasma concentrations of epinephrine and norepinephrine into an existing model of the HPA axis. Section 2 describes model development (Section 2.1), sensitivity analysis (Section 2.3), and parameter estimation (Section 2.4). Sections 3 and 4 discuss the results of the sensitivity analysis (Section 3.1) and parameter optimization with respect to data from a previous study [13] (Section 3.2).

## 2. Methods

In this section, we describe the development of a novel mathematical model integrating the HPA and SA axes. This proposed model includes stimulatory and inhibitory relationships between CRH, ACTH, cortisol, epinephrine, and norepinephrine as shown in Fig 1. To create subject-specific simulations from collected data, Morris screening and nonlinear optimization methods are described as they apply to the model parameters.

### 2.1. HPA-SA model

The proposed HPA-SA axis model is adapted from a model of the HPA axis by Bangsgaard and Ottesen [1]. This previous model was a system of ordinary differential equations (ODEs) representing the states of CRH, ACTH, and cortisol parameterized to patient-specific data to elucidate the cortisol response to stress in patients with clinical depression.

Our HPA-SA model builds upon the model from Bangsgaard and Ottesen with the addition of state ODEs for epinephrine and norepinephrine and several modifications to the HPA state equations:


dCdt=a0+Circ(t−θ)·(a11+a2B2)(Cμ+C)−ω1C (1)

$$\frac{dA}{dt}=\frac{a_{3}C}{1+a_{4}B}-\omega_{2}A$$ (2)

dBdt=a5A+a6E−ω3B (3)

dEdt=N(αeBNke+BN)−deE (4)

dNdt=αnS(t)kn+S(t)−αsBNks+BN−dnN, (5)

where where *C*, *A*, *B*, *E*, and *N* represent plasma concentrations of CRH, ACTH, cortisol, epinephrine, and norepinephrine, respectively. Eqs 1–3 in the HPA-SA model are based on the Bangsgaard and Ottesen model, with minor adaptations indicated by boxed terms [1]. Each equation is made up of production and elimination terms, which are positive and negative, respectively. Elimination encompasses multiple physiological processes, including metabolism and and excretion, that result in decreases in the plasma concentration of each hormone.

In Eq 1, production of CRH is driven by a basal rate (*a*_0_) in addition to the circadian rhythm (*Circ*(*t*)), which is multiplied by two Hill terms. The first term in Eq 2 represents the production of ACTH, which is stimulated by CRH (*C*) in the numerator and inhibited by cortisol (*B*) in the denominator. See Tables 1–2 for parameter descriptions. The function *Circ*(*t*) represents the 24-hour circadian rhythm, which influences the hormone secretion by the HPA axis in a periodic pattern, given by


$$Circ(t)=N_{c}\left(\frac{t_{m}^{k}}{t_{m}^{k}+\alpha^{k}}\cdot \frac{(T-t_{m}{)}^{l}}{(T-t_{m}{)}^{l}+\beta^{l}}+\epsilon \right),$$

**Table 1:** Parameters used in *S*(*t*).

Parameter	Description	Value
$\gamma_{min}$	minimum stress level	31 (AU)
$\gamma_{max}$	maximum stress level	121 (AU)
*ρ*	time of stress onset	10 (min)
*σ*	time of stress end	42 (min)
*κ*	steepness of stress increase	$0.2{\text{(min}}^{-1})$
*η*	steepness of stress decrease	$0.5{\text{(min}}^{-1})$
*τ*	time of peak stress	33 (min)

AU, arbitrary units.

**Table 2:** Parameter ranges for Morris screening.

Parameter	Description	(LB, UB)
*d*_*e*_	Elimination rate of E	$\left(3.90\times 10^{-6},3.90\times 10^{-4}\right)$
$\alpha_{e}$	Rate of production of E from N	$\left(6.84\times 10^{11},4.10\times 10^{14}\right)$
*d*_*n*_	Elimination rate of N	$\left(1.78\times 10^{7},1.78\times 10^{9}\right)$
$\omega_{3}$	Elimination rate of B	$\left(5.83\times 10^{1},5.83\times 10^{3}\right)$
*a*_6_	Stimulation rate of B by E	$\left(3.37\times 10^{-3},3.37\times 10^{-1}\right)$
*k*_*n*_	HS constant for stimulation of N by stress	$\left(1.90\times 10^{3},1.60\times 10^{7}\right)$
$\alpha_{n}$	Stimulation rate of N by stress	$\left(7.74\times 10^{2},1.77\times 10^{6}\right)$
$\alpha_{s}$	Rate of N loss via conversion to E	$\left(1.31\times 10^{-4},6.82\times 10^{1}\right)$
$\omega_{2}$	Elimination rate of A	$\left(1.82\times 10^{-10},1.38\times 10^{1}\right)$
*a*_5_	Stimulation rate of B by A	$\left(3.36\times 10^{-4},8.43\times 10^{1}\right)$
$\omega_{1}$	Elimination rate of C	$\left(3.4657\times 10^{-2},3.4657\times 10^{0}\right)$
*a*_2_	Negative feedback of B on C	$\left(2.888\times 10^{-2},2.888\times 10^{0}\right)$
*a*_4_	Negative feedback of B on A	$\left(9.91\times 10^{-11},3.03\times 10^{-2}\right)$
*a*_1_	Stimulation rate of C by circadian rhythm	$\left(4.00\times 10^{1},2.84\times 10^{4}\right)$
*μ*	HS constant for stimulation of C	$\left(1.70\times 10^{-3},9.35\times 10^{-1}\right)$
*a*_3_	Stimulation rate of A by C	$\left(1.50\times 10^{1},2.50\times 10^{2}\right)$
*k*_*e*_	HS constant for production of E from N	$\left(7.20\times 10^{1},1.44\times 10^{4}\right)$
*k*_*s*_	HS constant for conversion of N to E	$\left(6.00\times 10^{0},8.50\times 10^{2}\right)$
*a*_0_	Basal rate of production for C	$\left(5.00\times 10^{1},2.50\times 10^{3}\right)$

HS, half-saturation. LB, lower bound. UB, upper bound.

where $t_{m}\equiv \left(t-\delta \right)\;(\mathrm{mod}\;T),$ and *T* = 1440, representing the number of minutes in a day. The parameters of *Circ*(*t*) modulate the magnitude (*N*_*c*_, *ε*), half-sa*t*uration points (*α*, *β*), length (*T*), steepness (*l*,*k*), and time delay (*δ*) of the circadian and u*l*tradian rhythms.

Several adaptations have been made to Eqs 1–3. Firstly, the shift θ=490 minutes in the *Circ*(*t*) function aligns with the peak of *t*he circadian input with the approximate time that data collection started. Additionally, the first term of Eq 2 has been reduced from a second order to a first order term to be consistent with the addition of a first order (*a*_6*E*_) stimulatory effect from epinephrine on cortisol.

Eqns 4 and 5 were added to represent the dynamics of epinephrine and norepinephrine. Each of these equations is made up of production and elimination terms. The elimination terms are first order and incorporate the elimination rates *d*_*e*_ and *d*_*n*_. Eq 5 has an additional term, $-\frac{\alpha_{s}BN}{k_{s}+BN}$ which represents the loss of norepinephrine as it is converted to epinephrine via PNMT. A similar term is present in Eq 4 to represent the corresponding increase in epinephrine resulting from norepinephrine conversion. Lastly, the term $\frac{\alpha_{n}S(t)}{k_{n}+S(t)}$ represents the simulation of norepinephrine in response to the stress stimulus.

**Fig 2:**
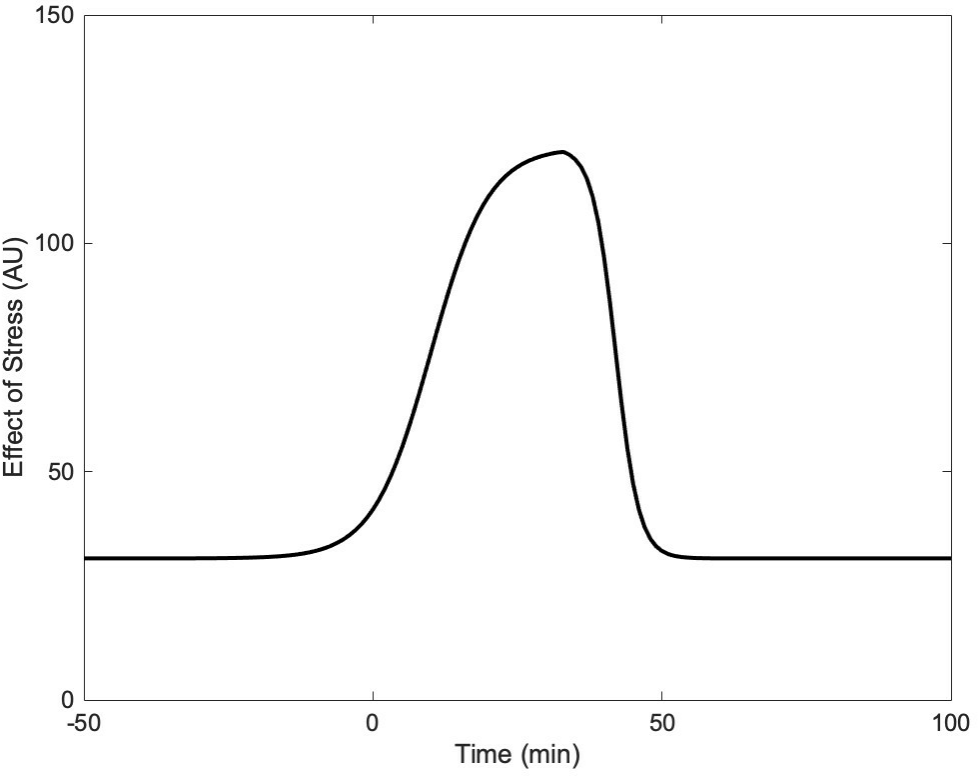
Stress as a function of time. Stress *S*(*t*) experienced by subjects over the course of the experimental protocol described in Section 2.2.

The piecewise sigmoidal function *S*(*t*), given by


$$\begin{array}{c} S(t)=\left\{ \begin{array}{cc} \gamma_{min}+\frac{\gamma_{max}-\gamma_{min}}{1+e^{-\kappa (t-\rho )}}, & 0\leq t\leq \tau \\ \gamma_{max}-\frac{\gamma_{max}-\gamma_{min}}{1+e^{-\eta (t-\sigma )}}, & t>\tau , \end{array}\right.\; \end{array}$$

represents the hypothesized gradual increase and decrease in stress experienced by subjects during the experimental protocol described in Section 2.2. The first sigmoid represents the increase in stress during exercise, while the second sigmoid represents the decrease in stress after the end of the exercise (see Fig 2). Each sigmoid was parameterized such that the steepness and the timing of the peak of *S*(*t*) would best match the stress protocol described in Section 2.2. Information about the stress function parameters can be found in Table 1.

The solutions to the system of ODEs for a time span of 150 minutes were obtained using the built-in function ode45 in MATLAB_R2024b [14]. Initial conditions were set equal to the first observation for each variable. The ode45 solver uses an adaptive time step. The solutions were then evaluated at each time for which there is a measurement. [13]

### 2.2. Data

**Fig 3:**
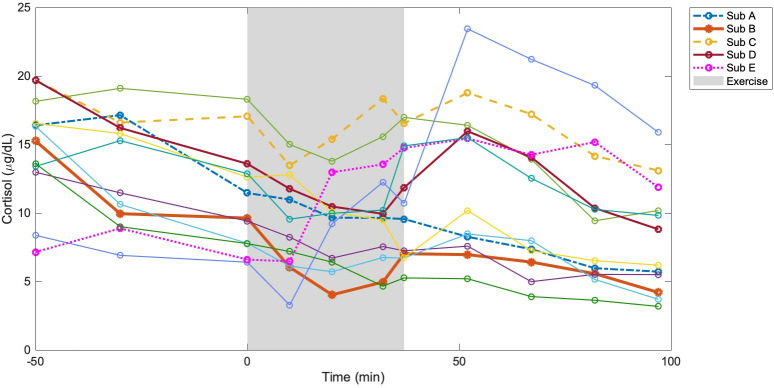
Cortisol data. Time series data for each subject’s cortisol concentrations measured by Webb et al. Each colored curve corresponds to an individual subject. Circles indicate measured values; shaded area indicates the duration of the exercise protocol. Subjects A-E are the five representative subjects included in Section 3.2.

The data used for initial parameter tuning (Section 2.3.1) and later optimization (Section 2.4) was collected as part of a study by Webb et al. examining the effects of different stress stimuli on hormone and catecholamine levels in firefighters [13]. In this experiment, subjects with healthy adrenal function cycled at 60% of their maximum oxygen consumption (VO2_max_) for a total of 37 minutes to simulate stress. Time series data for plasma concentrations of ACTH, cortisol, epinephrine, and norepinephrine were collected from each subject at 50, 30, and 0 minutes prior to exercise as well as at 10, 20, and 37 minutes during the exercise, and at 15, 30, 45, and 60 minutes after the exercise (during rest). Cortisol concentrations measured from each subject throughout the protocol are shown in Fig 3. The model was fit to five representative datasets.

### 2.3. Sensitivity analysis

#### 2.3.1 Preliminary Parameter Tuning.

The uncertainty of the parameter space was explored initially by overall tuning of parameters to fit the model to each subject data set. Initial parameter values were taken from Bansgaard and Ottesen [1] for parameters where available, and the remaining values were set such that the model outputs reasonably matched the data for ACTH, cortisol, epinephrine, and norepinephrine. These parameter values comprise the nominal parameter set for each subject and are found in S1 Table. Parameter ranges across the entire subject data set were used to inform a global sensitivity screening.

#### 2.3.2 Morris Screening.

Sensitivity analysis was performed on the entire set of parameters using a Morris screening algorithm. Morris screening utilizes elementary effects to quantify the influence of each parameter on the output of interest [15,16]. Elementary effects are calculated by


$$EE_{i}=\frac{f(X_{1},X_{2},...,X_{i}+\Delta ,...,X_{n})-f(X_{1},X_{2},...,X_{i},...,X_{n})}{\Delta },$$ (6)

where *X*_*i*_ is the *i*^th^ parameter in the parameter set, *Δ* is the step size, and $F(X_{1},...,X_{n})$ is the model output for the parameter set $\left\{X_{1},...,X_{n}\right\}$. The outputs of the Morris screening for each parameter are the mean (*μ*), mean of the absolute value ($\mu^{*}$), and standard deviation (*σ*) of its elementary effects over a set of random initializations of the parameter set. For *r* random initializations, these outputs are calculated by


$$\mu_{i}=\frac{\sum_{k=1}^{r}EE_{i}^{k}}{r}$$ (7)

$$\mu_{i}^{*}=\frac{\sum_{k=1}^{r}|EE_{i}^{k}|}{r}$$ (8)

$$\sigma_{i}=\sqrt{\frac{\sum_{k=1}^{r}(EE_{i}^{k}-\mu_{i}{)}^{2}}{r-1}}.$$ (9)

The mean values *μ* and $\mu^{*}$ characterize the effect of each parameter on the output of interest, while the standard deviation *σ* describes each parameter’s level of interaction with other parameters or nonlinearity [16]. Since each elementary effect can be either positive or negative for a non-monotonic output, $\mu^{*}$ is considered to be a more accurate measure of the mean elementary effect than *μ*. Further, the Morris index (MI), calculated as ${MI}_{i}=\sqrt{{\mu_{i}^{*}}^{2}+\sigma_{i}^{2}}$, gives a measure for the influence of each parameter on the model output by accounting for both the mean and variance, where a higher MI indicates a more influential parameter [15,16].

The parameter ranges used for Morris screening were based on the initial parameter tuning described in Section 2.3.1. These ranges are listed in Table 2.

We used 10,000 random initializations in order to adequately cover the large-magnitude parameter space and yield consistent Morris rankings with respect to the model output of interest (compare, e.g., with Colebank and Chesler or Stadt and Layton in which the smaller parameter space required only 100 or 1,000 runs, respectively). [16,17]

### 2.4. Optimization

Using the results from the Morris screening, the influential parameters were optimized from the nominal parameter set (see Section 2.3.1) using a built-in nonlinear constrained optimization function (fmincon). [14] Since the parameters represent physiological rates and quantities, they were bounded below by 0. To account for uncertainty around the parameter values, the parameters were each bounded above by $1\times 10^{3}$ times their nominal value. The objective when optimizing the parameters was to minimize the error for the associated state variables, *B*, *E*, and *N*. This error was calculated by


$$\sum_{i=1}^{n}{\left(\frac{\left(y_{i}-\hat{y_{i}}\right)}{\frac{1}{n}\sum_{i=1}^{n}y_{i}}\right)}^{2},$$ (10)

where *n* is the number of observations, *y*_*i*_ is the *ith* observation of state *y*, and $\hat{y_{i}}$ is the *ith* predicted value of state *y* for y∈{B,E,N}. As shown in Eq 10, the residuals were normalized by the mean of the observations. The error for CRH was not minimized due to lack of data for model comparison and calculation of residuals. The error for ACTH was not minimized in order to prioritize fitting the newer components of the model (see Eqs 4–5).

## 3. Results

We present results of the Morris screening and parameter optimization including simulated model outputs in comparison to the data for cortisol, epinephrine, and norepinephrine. These time series are plotted over the course of a 157-minute protocol, which included 50 minutes under normal conditions, 37 minutes under physical stress, and 60 minutes of recovery [13].

### 3.1. Sensitivity analysis

**Fig 4:**
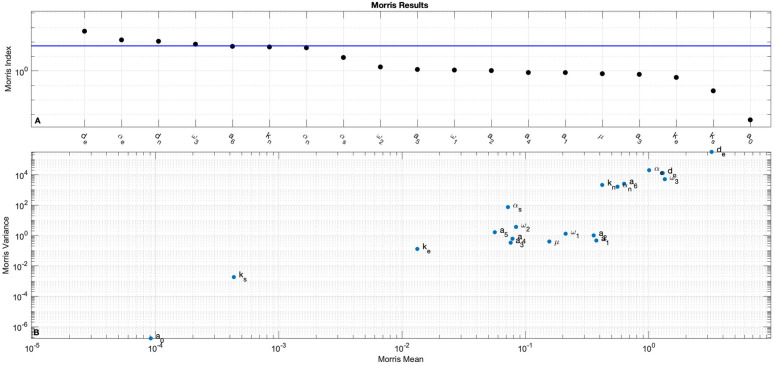
Morris screening outputs. A: Morris index (MI) for each of the 19 parameters. Blue line indicates the mean MI. B: Variance ($\sigma^{2}$) vs. Mean ($\mu^{*}$) for each of the 19 parameters.

The Morris indices for all 19 parameters with respect to maximum cortisol (B_max_) as calculated in Section 2.3 are shown in Fig 4A. While the mean ($\mu^{*}$) has been used in the past as a threshold for influential parameters, we see in Fig 4B that including parameters just below the mean leads to an influential cluster distinct from the rest of the parameters with lower Morris index: $\left\{d_{e},\alpha_{e},d_{n},\omega_{3},a_{6},k_{n},\alpha_{n}\right\}$, which are also the parameters with the seven highest MI values.

This set of parameters represents the elimination rate of epinephrine, production rate of epinephrine from norepinephrine, elimination rate of norepinephrine, elimination rate of cortisol, stimulation rate of cortisol by epinephrine, half-saturation constant for the stimulation of norepinephrine by stress, and stimulation rate of norepinephrine by stress, respectively. These parameters focus on the SA axis and cortisol. None of the influential parameters were directly associated with ACTH or CRH.

### 3.2. Parameter optimization

**Fig 5:**
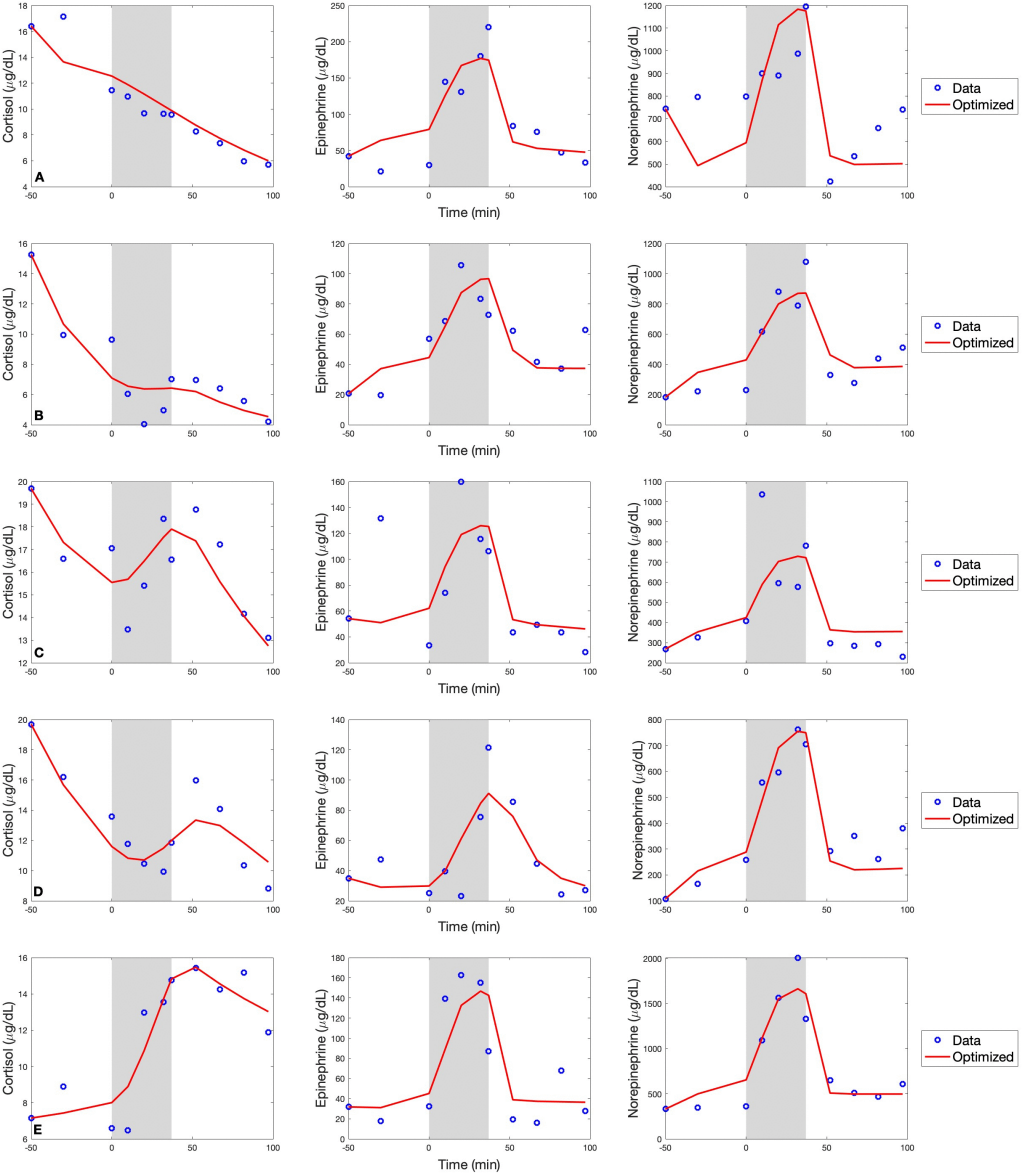
Simulations vs. data. Cortisol, epinephrine, and norepinephrine concentrations for 6 subjects after optimization of sensitive parameter set. The blue circles are the measured values from Webb et al. [13] and the red curve is the model solution with optimized parameters. The shaded region denotes the duration of the stress stimulus.

The influential parameters were optimized while keeping the remaining parameters fixed at their nominal values (see S1 Table). Fig 5 shows both the observed and simulated time series using optimized parameters for cortisol, epinephrine, and norepinephrine from five different subjects. Epinephrine and norepinephrine follow similar trends across subjects, while the dynamics of cortisol show more variation across subjects. In general, norepinephrine and epinephrine tend to maintain constant levels in the absence of stress, and consistently increase and decrease in accordance with the onset and termination of stress, respectively. While cortisol did not show such consistent behavior, its behavior was similar between some subjects. Specifically, subjects A and B showed similar decreasing behavior, while subjects C and D showed more fluctuating dynamics, and subject E shows a distinct increase over time.

The data and the simulated curve for Subject A both show near-monotonic decrease in cortisol. Subject A also had the highest estimated value for $\omega_{3}$, the elimination rate of cortisol. Although not strictly monotonic, Subject B also shows a decreasing trend in cortisol over time, and had the second highest estimated value for $\omega_{3}$ (see Table 3). When compared to the other three subjects, subjects C and D showed more fluctuation in cortisol during stress and had larger estimated values of *a*_6_, the rate of stimulation of cortisol by epinephrine (see Table 3). Subject E showed a marked increase in cortisol over the experimental protocol and had three parameters whose estimated values were higher than those of the other four subjects. These parameters were $\alpha_{n}$, the stimulation rate of norepinephrine by stress; *k*_*n*_, the half-saturation constant for the production of norepinephrine; and *d*_*n*_, the elimination rate of norepinephrine (see Table 3). Conversely, the estimated value of $\omega_{3}$ for subject E was lower than that of the other four subjects (see Table 3).

**Table 3:** Optimized values for influential parameters.

	Subject					
Parameter	A	B	C	D	E	Units
*d*_*e*_	12.896	2.1847	3.5967	0.061531	9.907	*min* ^-1^
$\alpha_{e}$	2.3781	0.2594	0.76022	0.009723	0.95055	–
*d*_*n*_	0.47144	0.2219	0.9375	0.39083	3.7505	*min* ^-1^
*ω*_3_	7.094	0.023672	0.021389	0.017534	0.011831	*min* ^-1^
*a*_6_	0.002326	0.001126	0.002605	0.003507	0.002533	–
*k*_*n*_	79.351	47.342	52.854	110.98	443.45	–
$\alpha_{n}$	1025.6	374.57	1096.4	725.65	29681.0	*μ*g/dL

Columns 2–6 list the optimized values of the seven influential parameters for subjects A-E, respectively. Each row gives the optimized values of a given parameter across the five different subjects.

Values of the error measure calculated by Eq 10 are shown for each subject in Table 4. Based on these error values, the model estimated the data best for subjects A, B, and D, while error values were higher for subjects C and E. Both subjects C and E exhibited higher levels of cortisol during and after the stress period compared to the other subjects.

**Table 4:** Error measures.

Subject	Error Measure
A	1.7929
B	1.6577
C	3.0696
D	1.7254
E	2.4628

Measure of error for subjects A-E as calculated by Eq 10.

The dynamics of epinephrine and norepinephrine observed in the data are reproduced by the model even without subject-specific parameterization of the stress function *S*(*t*). Despite the variability in cortisol across the five subjects, with the implementation of subject-specific optimization on the subset of influential parameters, the model gets reasonably close to the data, capturing the overall dynamics of each output over the course of the experimental protocol.

## 4. Discussion

The novel HPA-SA model developed in this study produced reasonable fits to data for plasma concentrations of cortisol, epinephrine, and norepinephrine. These fits captured general dynamics of model outputs B, E, and N across multiple subjects with a variety of observed behavior. While five representative datasets were shown, reasonable fits for B, E, and N were achieved for eleven datasets by performing Morris screening to determine the influential parameter set and using nonlinear optimization to estimate influential parameters based on available data.

The most influential parameters with respect to B_max_ are $\{d_{e},\alpha_{e},d_{n},\omega_{3},a_{6},$ $k_{n},\alpha_{n}\}$. All six of these parameters are associated with production and elimination of cortisol, epinephrine, and norepinephrine (see Table 2). These results from the Morris screening align with our understanding of the physiological response to short-term stress for which the catecholamines are the driving force. The least influential parameters with respect to B_max_ are $\left\{k_{e},k_{s},a_{0}\right\}$. *k*_*e*_ and *k*_*s*_ are half saturation constants for epinephrine and norepinephrine. The effect of these two parameters on the model outputs may be overshadowed by the effect of *S*(*t*). Additionally, since *a*_0_ is the basal secretion of CRH, and is not affected by stress, it is expected that this parame*t*er would not influence B_max_ during the observation period.

Data and simulated outputs for five different subjects are shown in Fig 5. These five subjects can be divided into three groups based on the trends in their measured cortisol concentrations over the course of the experiment, as described in Section 3.2. Each of these groups showed differences in parameter values that can be interpreted in terms of the relevant physiology. Subjects A and B show an overall decreasing trend in cortisol with higher values of $\omega_{3}$, the elimination rate of cortisol. This follows from the hypothesis that in order for cortisol to decrease during stress, it must be removed from the bloodstream at a higher rate. Subjects C and D showed more oscillatory behavior in cortisol along with a higher value for *a*_6_, the stimulation rate of cortisol by epinephrine. This may suggest that that short-term changes in cortisol are associated with increases in epinephrine. Subject E shows a significant, sustained increase in cortisol over the duration of stress with higher values for $\alpha_{n},k_{n},$ and *d*_*n*_, and a lower value for $\omega_{3}$. The first three parameters regulate the concentration of norepinephrine present. Lastly, a lower value for $\omega_{3}$ would allow for slower elimination of cortisol from the bloodstream and thus a more significant increase over time if cortisol is stimulated by stress. Table 4 shows that simulations for subjects C and D had the highest measure of error between predicted values and data. It appears that the large increases in cortisol during stress that were observed in these two subjects was one of the more challenging dynamics for the model to capture.

Limitations of this study come from multiple sources, including the available data and methods. Firstly, there are limitations associated with the data from Webb et al.’s study [13]. This study focused on exercise stress and therefore cannot be assumed to reflect the physiological effects of other types of stress such as psychological or social stress. Additionally, the subjects of the study cannot be assumed to be representative of the overall population. The subjects were all male firefighters, which may have different physiological stress responses when compared to other groups.

Secondly, there are limitations associated with the methods employed for parameter estimation and sensitivity analysis. The fmincon algorithm starts with nominal parameter values and searches the parameter space to locate a parameter set that minimizes the error as described in Section 2.4. However, the minimum error values determined by the algorithm are only guaranteed to be local minima and are therefore dependent on the nominal parameter values. Also, while sensitivity analysis was performed on the full parameter set, the sensitivity of the initial conditions for the five state variables was not considered in this study.

## 5. Conclusion

This HPA-SA model sets the stage for future mathematical models that can differentiate between healthy and unhealthy patient populations. Insights derived from further mathematical modeling efforts can increase our understanding of the behavior of the HPA and SA axes across subjects, which can in turn allow for the development of more effective ways of monitoring and treating disorders related to cortisol dysregulation.

## Supporting information

S1 TableNominal values for all parameters.Columns 2–6 list the nominal values of the parameters for subjects A-E, respectively. Each row gives the nominal values of a given parameter across the five different subjects. $\left\{d_{e},\alpha_{e},d_{n},\omega_{3},a_{6},k_{n},\alpha_{n}\right\}$ were optimized from these values (see Table 3), while the other parameters were fixed at their nominal values.(PDF)
